# Anatomy and Surgical Relevance of Rouviere's Sulcus

**DOI:** 10.1155/2013/254287

**Published:** 2013-11-06

**Authors:** Raja Dahmane, Abdelwaheb Morjane, Andrej Starc

**Affiliations:** ^1^Faculty of Health Sciences, University of Ljubljana, 1000 Ljubljana, Slovenia; ^2^Faculty of Medicine, Institute of Anatomy, University of Ljubljana, 1000 Ljubljana, Slovenia; ^3^Department of Surgery, Faculty of Medicine, University of the Center, 4011 Monastir, Tunisia

## Abstract

Rouviere's sulcus (RS) (i.e., incisura hepatis dextra, Gans incisura) represents an important anatomical landmark. The aim of the study was to determine the frequency of the RS, its description, its location, its relations to the right portal pedicle and to the plane of the common bile duct, and the evaluation of the surgical relevance of the obtained data. Forty macroscopically healthy and undamaged livers were removed during autopsies from cadavers of both sexes. The RS was present in 82% of the cases and in these the open RS was identified in 70% of the livers. The fused type was observed in 12% of the cases; 18% of the livers had no sulcus. The mean length of the open type RS was 28 ± 2 mm (range 24–32 mm) and its mean depth was 6 ± 2 mm (range 4–8 mm). The right posterior sectional pedicle was found in the RS in 70% of the cases. In 5% of the livers, we also dissected a branch of the anterior sectional pedicle. Inside 25% of the RS, we found the vein of segment 6. The RS identification may avoid bile duct injury during laparoscopic cholecystectomy and enables elective vascular control during the right liver resection.

## 1. Introduction

The knowledge of surgical anatomy is important for the safe execution of any surgical procedure. In the last decade, researchers have focused on many strategies to avoid complications during laparoscopic cholecystectomy [1, 2]. The common anatomical landmark or reference is Rouviere's sulcus (RS) [3–10]. Peti and Moser [11] determined that RS dissection is a lesser known, but important, anatomic work in every surgeon's strategy for safe cholecystectomy and avoidance of common bile duct injury, for safe laparoscopic cholecystectomy and the segment-oriented approach to right liver resection. The identification of this important landmark was done by Rouviere [12]. He used it as a reference point to guide the commencement of safe liver dissection [13–16]. Nevertheless, the RS, as a surgical landmark, is not widely used. There are nearly no data about it in the referential anatomical literature—its frequency is not well defined and its morphology is not exactly described. However, with the development of laparoscopic procedure, the surgical interest in the RS and its relation to the right portal pedicle has increased in recent years.

The aim of our study was to determine the frequency of the RS, its description, its relations to the right portal pedicle and to the plane of the common bile duct, and its relevance to techniques in liver surgery, particularly the laparoscopic cholecystectomy and the dissection of the right portal pedicle.

The terminology for liver subunits and liver surgery used in the present paper is in accordance with the Brisbane 2000 terminology [17].

## 2. Materials and Methods

In accordance with ethical and legal provisions, 40 macroscopically healthy and undamaged livers were removed during autopsies from cadavers of both sexes. The exclusion criteria were as follows: age lower than 18 years, death by abdominal trauma, chronic liver pathologies (cirrhosis and others), liver tumors discovered during the autopsies, and operated livers. On the removed liver, the inferior vena cava was ligated just before its entry into the right atrium; the serous and fatty tissue were cleaned off; frequency, location, and type of RS were documented. The length and width were measured. The open type of sulcus was defined as a cleft in which branches of the right hepatic pedicle were visualized and the sulcus was opened throughout its length. The frequency of parenchymatous fused type was measured, it was defined as the one in which the sulcus was open only in its lateral end [18]. Then, the plastic cannulas were inserted into the portal vein, the proper hepatic artery, and the common bile duct, which were to be injected to prepare the corrosive casts of the hollow structures of the liver. The resin polyester was used for injection; the injections were performed selectively. First the bile ducts and arteries requiring a small volume of the resin were injected and then the portal vein was injected as well. The preparations were then put into a 30% HCL solution. After a few days, they were rinsed with water jets, and the necrotic liver tissues were removed. The contents of the RS were determined.

*Statistics*. Data were expressed as means ± SD and ranges.

## 3. Results 

### 3.1. Frequency and Biometrics of the RS

**Figure 1:**
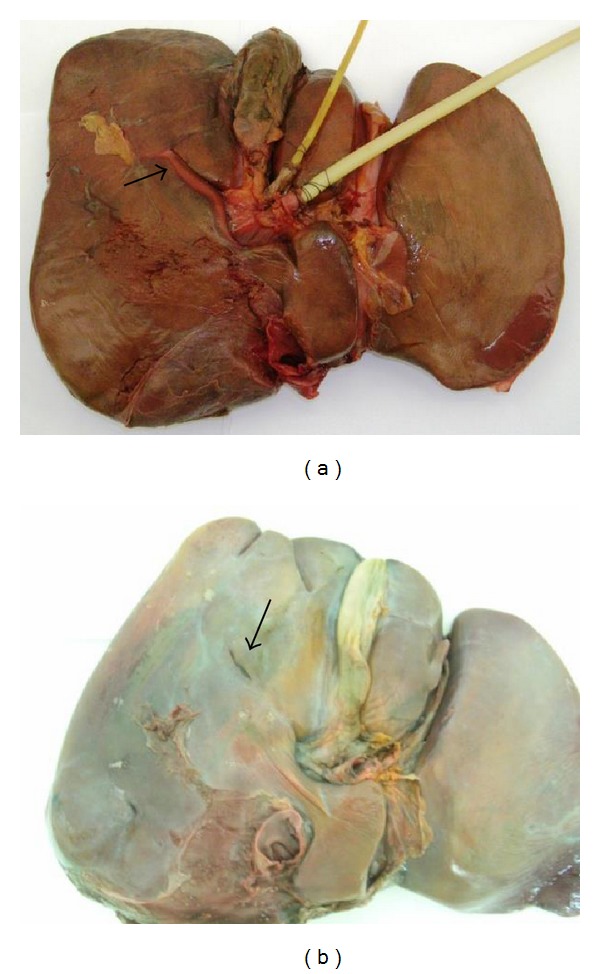
(a) Open type of Rouviere's sulcus with visible right portal pedicle. (b) Partially fused Rouviere's sulcus open at its lateral end.

The frequency of the RS was 82% and in these the open RS was identified in 70% of the livers (Figure 1(a)). The fused type was observed in 12% of the cases (Figure 1(b)). 18% of the livers had no sulcus.

The mean length of the open type RS was 28 ± 4 mm (range 24–32 mm) and its mean depth was 6 ± 2 mm (range 4–8 mm). 

### 3.2. Location and Orientation of the RS

**Figure 2:**
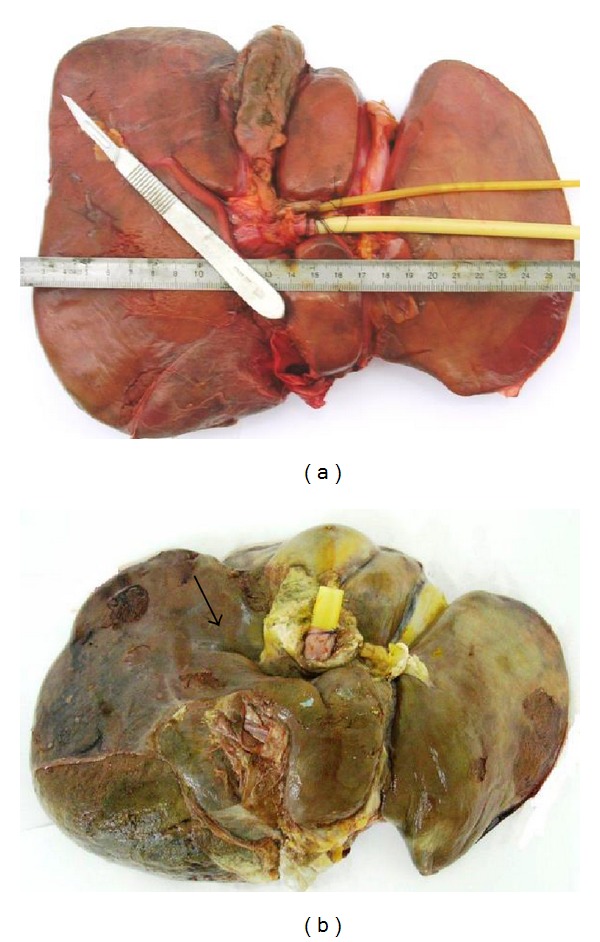
(a) Oblique type of Rouviere's sulcus. (b) Horizontal type of Rouviere's sulcus.

The RS is a cleft in the liver running to the right of the liver, anterior to segment 1. In 97% of the cases, it is oblique to the anterior, inferior, and external edge of the liver (Figure 2(a)), and in 3% of the livers it is horizontal (Figure 2(b)).

### 3.3. Contents of Rouviere's Sulcus

**Figure 3:**
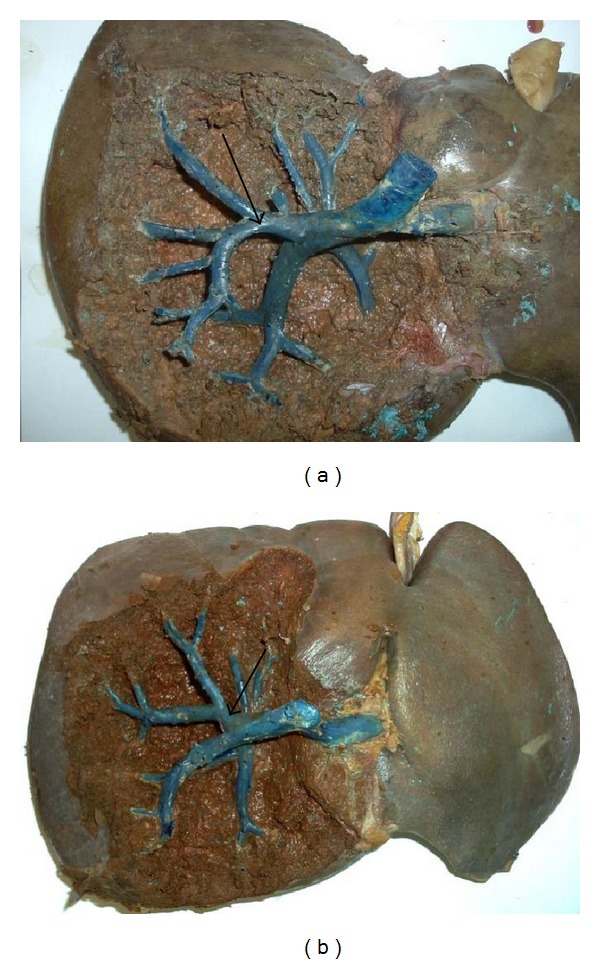
(a) Right posterior sectional pedicle in Rouviere's sulcus. (b) Right anterior sectional pedicle in Rouviere's sulcus.

The branches of the right posterior sectional pedicle were found in the RS in 70% of the cases. In 5% of the livers, we also dissected a branch of the anterior sectional pedicle. Inside 25% of the RS, we found the vein of segment 6 (Figures 3(a) and 3(b)). In 18%, we found the inconstant cystical vein. 

## 4. Discussion

The knowledge of liver anatomy and advances in imaging technology have the made operative procedure easier by reducing intraoperative bleeding and providing a low rate of postoperative complications. Anatomical variation and misidentification of the normal anatomical structures are major causes of surgical injury in laparoscopic procedure. Rouviere's sulcus is a 2-3 cm cleft running to the right of the liver hilum anterior to segment 1 and is usually containing the right portal triad or its branches. The sulcus indicates the plane of common bile duct accurately.

### 4.1. Terminology

In the surgical and anatomical literature, different names for the RS can be found. This cleft of the liver has been described as Incisura Dextra of Gans, by Reynaud, Coucoravas, and Giuly et al. [19] and subsequently also by Stringer in “Eponyms in Surgery and Anatomy of the Liver, Bile Ducts and Pancreas” [20]. Rouviere [12] was the first to name it “le sillon du processus caudé” In surgical anatomy, it is known as Rouviere's sulcus. 

### 4.2. Anatomical and Surgical Relevance

Most of the classic anatomical literature [21, 22] does not include data on the RS. Gans [23] described the RS in 80% of the livers. Couinaud [24] reported it as a very inconstant structure. Rouvière and Delmas [25] described the sulcus of the processus caudatus as a profound cleft between the renal and the duodenal impression, anterior to the processus caudatus. Reynaud et al. [19] observed the Incisura Dextra of Gans in 73% of the cases. RS has been found by Hugh et al. [26] in 78% of the livers and by Zubair et al. [18] in 68% of their cases.

The most important advantage of identifying RS lies in the fact that the cystic duct and the cystic artery lay anterosuperior to the sulcus [18] and the common bile duct lays below the level of the RS [11]. Hugh [3] had shown minimal common bile duct injury during laparoscopic cholecystectomy by beginning the dissection ventral to the RS.

The second technical reason for identifying RS is to perform safe right sectional or segmental liver resections. The fibrous sheath of Glisson encircles the hepatic artery, portal vein, and bile duct at the hilum and continues as the liver capsule. Liver resection of segments 6 and 7 is feasible with no major difficulties because the Glissonian sheaths of these segments pedicles present early bifurcation near the hilar plate and their course may be apparent inside the RS [7]. In the resection of segment 5, cholecystectomy is performed first, ventral to RS to avoid injury to the right posterior sectional pedicle [27]. 

## 5. Conclusions

Rouviere's sulcus is a frequent anatomical landmark present in 82% of the livers, either as open or fused type. Its identification may help avoid bile duct injury during laparoscopic cholecystectomy and enables elective vascular control during segment-oriented approach to right liver resection.
